# Random-phase metasurfaces at optical wavelengths

**DOI:** 10.1038/srep28448

**Published:** 2016-06-22

**Authors:** Anders Pors, Fei Ding, Yiting Chen, Ilya P. Radko, Sergey I. Bozhevolnyi

**Affiliations:** 1 SDU Nano Optics, University of Southern Denmark, Campusvej 55, DK-5230 Odense M, Denmark; 2 Department of Physics, Technical University of Denmark, DK-2800 Kongens Lyngby, Denmark

## Abstract

Random-phase metasurfaces, in which the constituents scatter light with random phases, have the property that an incident plane wave will diffusely scatter, hereby leading to a complex far-field response that is most suitably described by statistical means. In this work, we present and exemplify the statistical description of the far-field response, particularly highlighting how the response for polarised and unpolarised light might be alike or different depending on the correlation of scattering phases for two orthogonal polarisations. By utilizing gap plasmon-based metasurfaces, consisting of an optically thick gold film overlaid by a subwavelength thin glass spacer and an array of gold nanobricks, we design and realize random-phase metasurfaces at a wavelength of 800 nm. Optical characterisation of the fabricated samples convincingly demonstrates the diffuse scattering of reflected light, with statistics obeying the theoretical predictions. We foresee the use of random-phase metasurfaces for camouflage applications and as high-quality reference structures in dark-field microscopy, while the control of the statistics for polarised and unpolarised light might find usage in security applications. Finally, by incorporating a certain correlation between scattering by neighbouring metasurface constituents new types of functionalities can be realised, such as a Lambertian reflector.

Light-matter interaction is of fundamental importance in any branch of science or application that expresses a need to generate, control, or detect an electromagnetic wave. With the birth of nanotechnology, particularly the ability to structure matter on the nanoscale, the study of light-matter interaction has in the last decades attracted considerable attention from researchers and engineers due to new and unforeseen ways of manipulating light by nanostructuring material on a subwavelength scale. As pivotal achievements, illustrating the new degrees of light control, we mention negative refraction^1^,^2^, invisibility cloaking^3^,^4^, and super resolution^5^. These and many other applications are conceptually realized by the interaction of light with metamaterials^6^ or (the two-dimensional analogue) metasurfaces^7^,^8^,^9^ featuring effective responses unattainable with conventional materials or interfaces. Particularly metasurfaces, with their low profile, ease of fabrication, and relatively low losses, have in recent years gained noticeable attention, as they represent realistic components in future real-world applications. However, in fully controlling light with metasurfaces, it is required that one can engineer, both spectrally and spatially, the amplitude and phase of either the transmission or reflection coefficient at will, preferably for two orthogonal polarisations simultaneously. One configuration that to a large extent achieves this property is the generalization of the reflectarray concept from the low-frequency regime^10^, which, as the name suggests, works in reflection and consists of an optically thick metal film overlaid by a subwavelength thin dielectric spacer and an array of metal nanobricks (also known as nanopatches) separated by subwavelength distances. Since this type of metasurface has been realized in practically all frequency regimes it goes by many names, but in the visible and infrared regimes they are typically named meta-reflectarrays^11^, film-coupled nanoantenna metasurfaces^12^, or, as used in this work, gap plasmon-based metasurfaces, hereby highlighting the crucial role of gap surface plasmon (GSP) resonances in achieving the control of light^13^,^14^. GSP-based metasurfaces have been designed for a very diverse range of applications, ranging from broadband absorbers^15^,^16^ and flat optical components^17^,^18^,^19^, via polarisation-controlled unidirectional excitation of surface plasmon polaritons^19^ and efficient holograms^20^,^21^, to analogue light processing^22^, fast detection of light’s state of polarisation^23^, and cloaking^24^. The common denominator of all these applications is the well-defined reflection coefficient that is implemented on the metasurface by proper positioning and dimensioning of the nanobricks, hereby leading to, one might say, a deterministic response in the sense that no random variables enter the design of the metasurface. In contrast, recent work^25^,^26^,^27^,^28^,^29^ on reducing the radar cross section in the giga- and tera-hertz regimes, particularly relevant for stealth technology, has proposed reflectarrays featuring a (quasi-)random distribution of reflection phases, which will result in destructive interference of the scattered light, thus leading to a diffusion of light into a speckle-like far-field pattern and an overall low reflection. The performance of the metasurface is by design angle insensitive, and by utilizing four-fold symmetric unit cells one also achieves a polarization insensitive response. It should be noted that the best performance with respect to reflection reduction in a spectrally broad window is not a fully random-phase metasurface, as employment of optimisation routines suggests a certain correlation between neighbouring unit cells^25^,^26^,^27^. In relation to radar cross section reduction, we also note earlier work utilizing a random positioning of *π*-phase different elements which results in a chessboard-like surface^30^,^31^, and related (though different) work on the subject of cloaking by scattering cancellation^32^. Also, it is appropriate to mention a recent theoretical work on the estimation of the level of diffuse scattering in metasurfaces featuring small (but random) fluctuations in the unit cell properties^33^. Finally, we would like to highlight past work on characterising and understanding the influence on metasurface properties when an initial periodic arrangement of constituents becomes progressively disordered or completely amorphous^34^,^35^,^36^.

In this work, we present and discuss the far-field statistics of truly random-phase metasurfaces, particularly focusing on the probability density function (PDF) of the scattered intensity and how the metasurface response for polarised and unpolarised light can be alike or different depending on the correlation between orthogonally polarised scattering phases. By exploiting the large phase response from GSP-based metasurfaces, we design and realize random-phase metasurfaces that work around the wavelength of 800 nm. Optical characterisation of the fabricated metasurfaces confirm low-reflection and diffuse scattering of light with statistics following the theoretical predictions. As such, random-phase metasurfaces at optical wavelengths represent a diffuse scattering surface that might find application within camouflage technology and as a high-quality reference structure in reflective dark-field microscopy measurements. Moreover, the possibility for different statistics for polarised and unpolarised light might find usage in security applications. Finally, we foresee that incorporation of a certain correlation between scattering phases of neighbouring elements might lead to new functionalities, such as a sub-wavelength thin Lambertian reflector.

## Results

### Statistics

**Figure 1:**
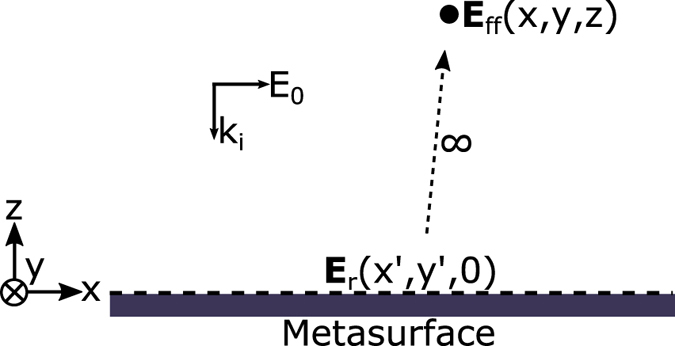
Sketch of metasurface configuration. The metasurface is positioned at *z* = 0 and interacts with a plane incident wave propagating along the −*z*-axis. The reflected electric field at the metasurface **E**_r_ is related to the scattered far-field **E**_ff_ via a two-dimensional spatial Fourier integral.

In the following discussion we consider a plane metasurface positioned at *z* = 0, with the incident light being represented by a plane wave propagating along the −*z*-direction (see Fig. 1). As the light interacts with the metasurface, part (if not all) of the electromagnetic field will be reflected, hereby resulting in a particular far-field in the upper medium (*z* > 0) that is determined by the properties of the metasurface. The reflected electric near-field at the metasurface **E**_r_ is related to the far-field counterpart **E**_ff_ via a two-dimensional spatial Fourier integral [time convention: exp(−*iωt*)]


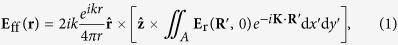


where *k* is the wave number, *r* is the distance from the origin to the point of observation **r** = (*x*, *y*, *z*), 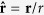 is the unit vector in the direction of **r**, *A* is the area of the metasurface, **R**′ = (*x*′, *y*′) is the in-plane coordinates at the metasurface, and **K** = (*k*_*x*_, *k*_*y*_) = *k*(*x*/*r*, *y*/*r*) is the in-plane wavevector. It should be noted that the near-field to far-field transformation in equation (1) is based on an alternative form of the field equivalence principle, which is defined entirely by the electric near-field and can be derived from the angular spectrum representation of the electromagnetic field^37^.

We now consider an isotropic metasurface whose reflection coefficient can be written as


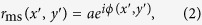


where the reflection amplitude *a* is constant along the metasurface, while the spatially dependent reflection phase *ϕ* is a *random variable* described by a uniform probability distribution


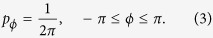


As an example, if we assume the incident plane wave to be *x*-polarised with the amplitude *E*_0_, the reflected near-field is 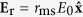 and the associated electric far-field [equation (1)] takes the form


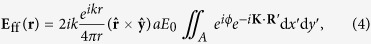


where the integral represents a sum of many random complex numbers. In other words, from the central limit theorem of statistics it follows that the electric far-field approximates a complex Gaussian random variable, while the statistical independence between reflection phases of neighbouring points on the metasurface allow us to describe the resultant electric far-field as a *random walk* with a constant step size (*aE*_0_) but an unknown direction (*ϕ*) for each step. Such a process features a probability density function (PDF) for the intensity that obeys negative exponential statistics^38^, i.e.,


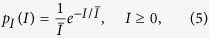


where 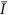 is the mean intensity. As expected from the independence between the contributions comprising the resulting far-field, the statistics of random-phase metasurfaces are equivalent to that of speckle patterns originating from a rough (on a wavelength scale) surface, but actually also polarised thermal light shows this kind of statistics^38^. Moreover, it is important to note that the removal of specular reflection by random-phase metasurfaces, together with the fact that the intensity value with largest probability is zero, are the reasons why these surfaces are interesting for stealth technology, as they represent typical scattering from the empty atmosphere and, hence, in some sense may be viewed as a simple alternative to true cloaking configurations^24^,^32^. It should also be kept in mind that equation (5) simultaneously represents the statistics of the far-field intensity in one specific direction measured over many metasurfaces and, due to the statistical independence between the intensity of different far-field directions, the statistics of the entire far-field for a single metasurface.

In the above discussion we assumed the metasurface to be isotropic, meaning that the reflection phases for two orthogonal polarisations are completely correlated. This has the consequence that equation (5) also represents the statistics of the reflected intensity for *unpolarised* light. If, however, the metasurface is anisotropic and described by a diagonal (2 × 2) reflection matrix, where the random reflection phases in a given point (*x*′, *y*′) for the two orthogonal polarisations are statistically independent, the statistics of the far-field intensity should for unpolarised incident light be described by a convolution of the PDFs for the two polarisation states [i.e., equation (5)], thus resulting in


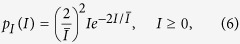


which is the same probability as for unpolarised thermal light^38^. Interestingly, this kind of PDF features a maximum at 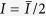, which is very different from the negative exponential statistics of reflected light for polarised incident light.

### Numerical examples

In order to gauge the statistics of ideal random-phase metasurfaces that are comprised of periodically arranged nanostructures, we present simple numerical calculations of the electric far-field when each unit cell reflects light with a certain phase. Based on equation (4), the underlying equation for *x*-polarised incident light takes the form


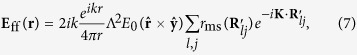


where 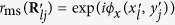 describes a perfect reflector with random reflection phase *ϕ*_*x*_, 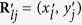 is the in-plane coordinates, Λ is the width of the square unit cell, and the sum includes contributions from all the unit cells comprising the fictive metasurface. It should be noted that the far-field expression for *y*-polarised light can be obtained by substituting 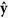 with 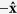 in equation (7). In the calculations to be presented, we choose a wavelength of *λ* = 800 nm and the surrounding medium to be air, while the array consists of 200 × 200 unit cells with inter-particle spacing of Λ = 250 nm, which are typical parameters for visible and near-infrared metasurfaces.

**Figure 2:**
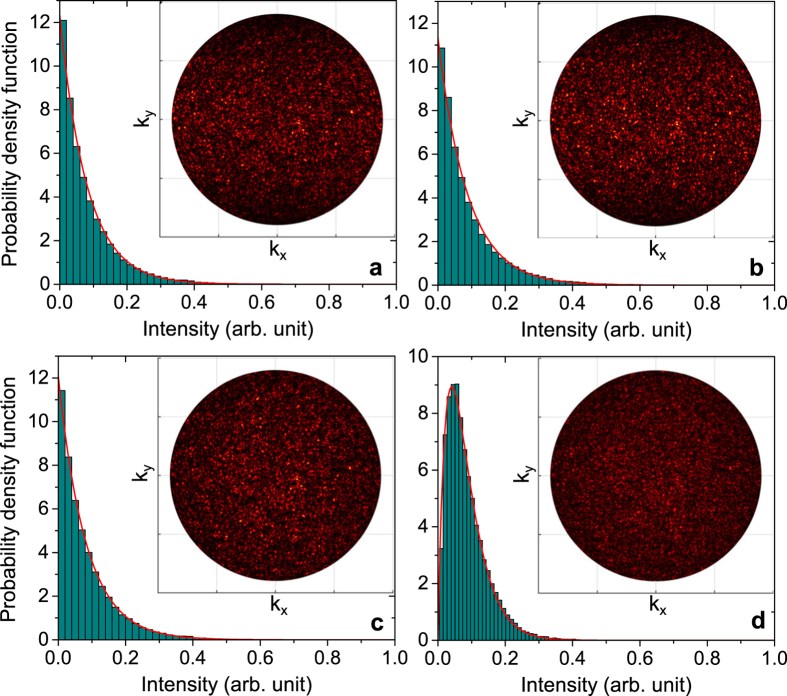
Numerical modelling of scattering from ideal random-phase metasurfaces. Probability density function of the far-field intensity from an array of 200 × 200 unit cells for excitation by (**a**) *x*-polarised incident light when unit cells feature random phases described by equation (3); (**b**) *x*-polarised incident light when the random phases of the unit cells are represented by a discrete random variable that takes on values (0, *π*/2, *π*, 3*π*/2), each with the probability *p*_*ϕ*_ = 1/4; (**c**) unpolarised incident light when the phases for orthogonal reflection are completely correlated [i.e., 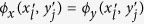] and described by equation (3); (**d**) unpolarised incident light when both *ϕ*_*x*_ and *ϕ*_*y*_ are described by equation (3) but are statistically independent. The histogram plots show the PDF of the calculated far-field intensity in the upper half-space within an NA of 0.9, while the red lines correspond to the theoretical predictions by equations (5) and (6). The insets show the associated Fourier images of the far-field intensities within the NA = 0.9.

As a way to mimic the scattering from a random-phase metasurface for *x*-polarised incident light, we calculate the far-field intensity through the relation *I*∝|**E**_ff_|^2^ (see the Methods section for details). The inset of Fig. 2a shows the far-field intensity calculated on a hemi-sphere in the upper half-space covering the polar angles [0; sin^−1^ (*NA*)] with NA = 0.9, which is equivalent to the experimental situation of recording a Fourier image of the reflected light from a metasurface with a lens of numerical aperture (NA) of 0.9. It is evident that the far-field resembles a typical speckle pattern, which is also confirmed by the calculated PDF (Fig. 2a) that convincingly follows the theoretical prediction of equation (5). In regard to these calculations, it is important to notice that representing the reflection phase *ϕ*_*x*_ by a continuous random variable covering the whole 2*π* phase space is an idealization, since lossy metasurfaces typically never cover the full phase space and, for this reason, a finite number of phases are used in the design in order to equally represent all parts of the 2*π* phase. In order to consider the effect of *ϕ*_*x*_ being a discrete random variable, Fig. 2b displays the far-field intensity and PDF when 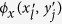 only takes on one of the four values (0, *π*/2, *π*, 3*π*/2), each value represented by the probability *p*_*ϕ*_ = 1/4. As evident, the quite rough discretisation of the phase space has minimal influence on the resulting far-field, with equation (5) still accurately describing the statistics of the intensity. Also, the amount of light being reflected within the NA is 26.6% and 26.5% for the metasurface featuring a complete random phase and four-phase response, respectively, thus confirming the low-reflection property and equivalent performance of both metasurfaces.

In regard to many practical applications, it is also important to gauge the scattering from random-phase metasurface for unpolarised incident light. Here, we investigate two extreme situations: (i) the random phases for orthogonal polarisations are completely correlated, i.e., 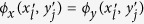, and ii) the phases are statistically independent. The latter case represents the situation where *ϕ*_*x*_ and *ϕ*_*y*_ are specified independently of each other. In accordance with the discussion in previous section, the statistic of the far-field intensity for the two cases is very different (see Fig. 2c,d), with the first case showing the same PDF as for polarised light, while the second case demonstrates a scattering response as predicted by equation (6). We note that the reflectivity in both cases is 26.6%, but it is the redistribution of the incoming energy that is different. The calculations of Fig. 2c,d showcase the important result that metasurfaces can be engineered to show different statistics of the reflected/transmitted light depending on the incident light being polarised or unpolarised. We foresee that such a property might be of interest in security applications.

### Gap plasmon-based metasurfaces

**Figure 3:**
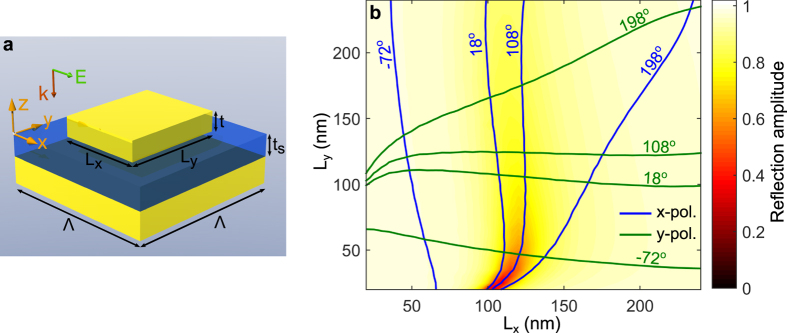
Reflection from GSP-based metasurfaces. (**a**) Drawing of the unit cell in a GSP-based metasurface, consisting of a dielectric spacer sandwiched between an optically thick metal film and an array of metallic nanobricks with subwavelength periodicity. (**b**) Calculated reflection coefficient as a function of nanobrick widths for a gold-SiO_2_-gold configuration with geometrical parameters *t* = *t*_*s*_ = 40 nm and Λ = 250 nm at a wavelength of 800 nm. Colour map shows the reflection coefficient amplitude for *x*-polarised normal incident light, while lines are contours of the reflection phase for both *x*- and *y*-polarisation.

In the remaining part of this work, we concentrate on the realization of random-phase metasurfaces that work in reflection at optical and near-infrared wavelengths. As a natural continuation of our previous work, we look for a solution by using GSP-based metasurfaces, which consist of an optically thick metal film overlaid by a nanometer thin dielectric spacer and an array of metal nanobricks with subwavelength periodicity (Fig. 3a). This type of metasurface has the advantage that it can be fabricated in one step of electron beam lithography, while it facilitates the possibility to control both the amplitude and phase of the reflected light for one polarisation^22^,^39^ or to simultaneously engineer the phases of two orthogonal polarisations^13^. In the following discussion, we choose the metal and spacer layer to be gold and silicon dioxide, respectively, with the spacer and nanobrick thicknesses being *t* = *t*_*s*_ = 40 nm. The size of the unit cell is Λ = 250 nm. As a customary procedure, valid within the local periodicity approximation^10^, inhomogeneous metasurfaces are constructed from the optical response of the homogeneous counterparts. Figure 3b shows a colour map of the reflection coefficient amplitude from a homogeneous GSP-based metasurface as a function of nanobrick widths (*L*_*x*_, *L*_*y*_) when the normal incident plane wave is *x*-polarised and the design wavelength is 800 nm. Superimposed on the colour map are contour lines of the reflection phase (in steps of 90°) for both *x*- and *y*-polarisation. It is evident that as *L*_*x*_ is increased (keeping *L*_*y*_ fixed) the reflection amplitude will feature a dip with a simultaneous strong variation in the reflection phase, hereby allowing one to cover up to ~90% of the 2*π* phase space by proper choice of nanobrick dimensions. As discussed in depth elsewhere^13^, the dip in the reflection amplitude is the signature of the excitation of the fundamental gap-plasmon resonance, also sometimes referred to as the magnetic resonance^40^. In this work, we would like to control the reflection phases of orthogonal polarisations *independently*, which graphically transpires to the requirement that phase contour lines for the discretised phase values must intersect each other. For this reason, we are limited to discretise the reflection phase in steps of 90°, which, c.f. Fig. 3b, ensures that each contour line for *x*-polarisation intersects the four contour lines for *y*-polarisation and vice versa. The sixteen nanobrick dimensions, defined by the contour line intersections, constitute a set of nanostructures that can be used (by random positioning in the array) to realize a random-phase metasurface for which the local reflection phases for orthogonal polarisations are statistically independent, hence featuring the PDF of equation (6) for unpolarised light. Similarly, the four nanobrick dimensions, as defined by the phase contour line intersections along the line *L*_*x*_ = *L*_*y*_, comprise a set of elementary unit cells that by random positioning in an array will feature completely correlated local reflection phases and, hence, the scattered light from both polarised and unpolarised light will feature the PDF of equation (5).

**Figure 4:**
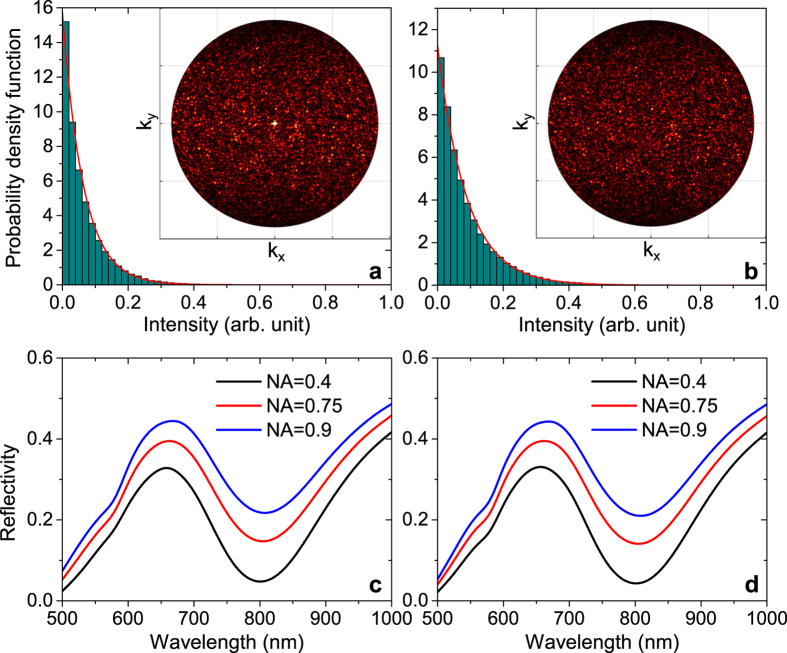
Numerical modelling of scattering from random-phase GSP-based metasurfaces. (**a**) Calculated (histogram) and theoretical (red line) PDF of the far-field intensity (when neglecting specular reflection) from an array of 200 × 200 unit cells for excitation by *x*-polarised incident light when the four types of nanobricks, defined by the intersection of contour lines in Fig. 3b for *L*_*x*_ = *L*_*y*_, appear with equal probability in the array. The inset shows the associated Fourier image of the far-field intensity within the NA = 0.9. The image is oversaturated in order to visualise the diffusion of light. (**b**) Similar calculation as in **a**, but the frequency of occurrence of the four nanobricks is scaled by the inverse of their reflection amplitudes. (**c**,**d**) Effective reflectivity (i.e., limited by the NA) as a function of wavelength and NA for the metasurfaces described in (**a**,**b**) respectively.

In the above discussion of realizing random-phase metasurface (with different statistics), we have ignored the fact that the nanobricks constituting the metasurfaces do not scatter light with equal strength, as evident from the variation in the reflection amplitude on the nanobrick dimensions (Fig. 3b). In order to illustrate the impact of the unequal scattering by nanobricks on the far-field response, we resort to similar calculations as in the previous section, though this time the reflection amplitude 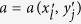 is also position-dependent, with values taken from the full-wave simulations (Fig. 3b). Here, we limit the discussion to the case of *x*-polarised incident light interacting with the random-phase metasurface comprised of four different nanobricks (whose reflection coefficients correspond to the intersections defined by *L*_*x*_ = *L*_*y*_ in Fig. 3b), but the calculated scattering for unpolarised light and the optical response of the metasurface featuring sixteen different nanobrick unit cells can be found in Supplementary Figs 1 and 2, respectively. The resulting far-field response in the upper half-space is shown in the inset of Fig. 4a, clearly signifying that the unequal scattering strength leads to significant specular reflection that on a linear scale overshadows the speckle-like scattering pattern of the remaining space. The high intensity in the the specular direction, of course, modifies the intensity statistics of the whole far-field, as it now becomes more probable to measure a high intensity value, which is in contrast to equation (5) stating that such a measurement is very unlikely to occur. Nevertheless, if we disregard the specular reflection, it is evident that the remaining far-field response follows negative exponential statistics (Fig. 4a) similar to the case of constant reflection amplitude (Fig. 2b). Moreover, we would like to emphasize that only a small part of reflected light is contained in the specular light, which is expressed in the reflectivity changing from 21.6% to 21.2% when the centre spot in the inset of Fig. 4a is excluded in the calculation. Finally, it is appropriate to mention that the specular reflection can indeed be removed in a rather simple way by modifying the probability of occurrence of the individual nanobrick unit cells in the array by a factor proportional to the inverse of their reflection amplitude. This fact is illustrated in Fig. 4b, where the whole half-space features a speckle-like pattern obeying the statistics of equation (5).

As the operation bandwidth of any metasurface, in general, is a crucial parameter for its potential success, we have calculated the wavelength-dependent (and NA-limited) reflectivity of the two GSP-based random-phase metasurfaces in Fig. 4a,b within the range 500–1000 nm. The spectral dependence of the unit cell reflection coefficients is extracted from full-wave simulations, with the resulting effective reflectivity from the random-phase metasurfaces [based on equation (7)] displayed in Fig. 4c,d for three different NAs. It is evident that the two metasurfaces feature, as expected, a minimum in reflection at the design wavelength *λ* = 800 nm, which owes to the optimal destructive interference of the scattered light. The low-reflecting property is, however, broadband, demonstrating a considerable suppression of reflection within the range 700–900 nm. Finally, it is worth noting that the practically identical reflection from the two metasurfaces, together with the significant change in detected reflection signal as a function of NA, signifies that efficient diffusion of light (with only a minor part of the reflected intensity being contained in the specular spot) also occurs away from the design wavelength.

### Experiments

With the above numerical results illustrating the statistics of the scattered light from random-phase metasurfaces, including a design based on GSP-based metasurfaces, we now proceed with the experimental verification of such metasurfaces. The random-phase metasurfaces are, c.f. Fig. 3b, realized by random positioning of the four or sixteen different nanobrick elements, thus representing diffusive surfaces with correlated or statistically independent reflection phases for orthogonal polarisations. The metasurfaces cover an area of 75 × 75 *μ*m^2^ and are fabricated using standard deposition techniques and electron-beam lithography, with the nominal sizes and random positioning of nanobricks being identical to that of the numerical simulations (see the Methods section for details). Moreover, it should be noted that in these proof-of-concept experiments we do not try to compensate for the different reflection amplitudes of the nanobricks, since a successful suppression of all specular reflected light requires precise knowledge of the optical properties of the deposited materials, a requirement not typically met for evaporated gold^41^.

**Figure 5:**
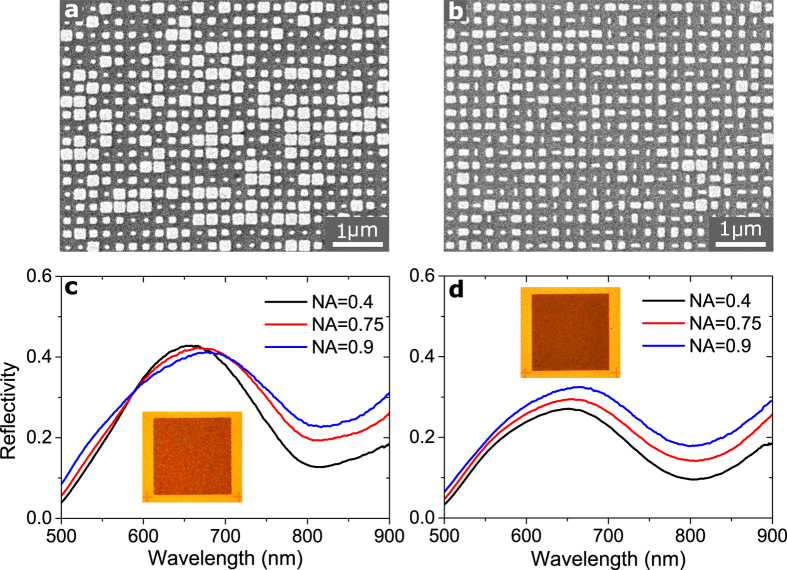
Reflection from random-phase GSP-based metasurfaces. (**a**,**b**) Representative scanning electron microscopy images of the fabricated GSP-based metasurfaces featuring 4 and 16 different nanobrick elements, respectively, thus approximating random-phase metasurfaces with fully correlated and statistical independent reflection phases for orthogonal polarisations. (**c**,**d**) Measured reflectivity as a function of wavelength and NA for the metasurfaces in (**a**,**b**) respectively. The incident light is *x*-polarised. The insets are bright-field images of the metasurfaces and surrounding gold film.

Figure 5a,b display representative images of the two types of fabricated metasurfaces, demonstrating in both cases that the nanobricks are properly resolved, though not perfectly square or rectangular. Particularly, the deviation from perfect squares in the four-element metasurface (Fig. 5a) immediately suggests that orthogonal reflection phases are not perfectly correlated. In order to probe the overall performance of the fabricated metasurfaces, we have measured the reflectivity for *x*-polarized incident light as a function of wavelength and NA (Fig. 5c,d). It is evident that the measured reflectivity from both metasurfaces qualitatively agrees with the numerical predictions (see, e.g., Fig. 4c), exhibiting a low reflection in the entire wavelength range 500–900 nm and a clear minimum at the design wavelength of 800 nm, which is due to the destructive interference of the scattered light. The broadband low-reflection property of both metasurfaces is also immediately clear from the corresponding bright-field images (see insets of Fig. 5c,d). Moreover, the overall measured reflection signal increases with the NA of the objective, thus verifying significant diffusion of reflected light. It should be noted that the increase in the measured reflectivity as a function of NA is not as significant in experiments as in simulations, which we ascribe to a non-ideal metasurface performance arising from fabrication imperfections and uncertainties in material properties.

**Figure 6:**
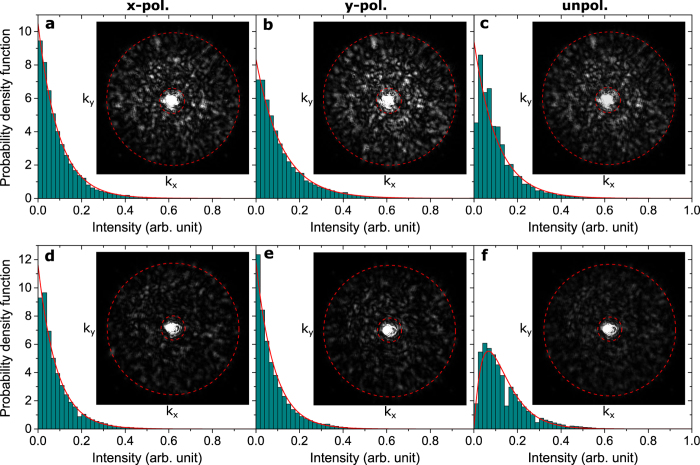
Statistics of scattered light. Measured (histogram plot) and theoretical (red line) PDF of the reflected far-field intensity (when neglecting specular reflection) from GSP-based metasurfaces with (**a**–**c**) fully correlated and (**d**–**f**) statistical independent reflection phases for orthogonal polarisations. The wavelength of the incident light is 800 nm and it is either (**a**,**d**) *x*-, (**b**,**e**) *y*- or (**c**,**f**) un-polarised. The insets show the associated Fourier images of the far-field intensity within the NA = 0.55, with the area bounded by the dashed circles indicating the scattered light used for the statistics. It should be noted that the Fourier images in (**c**,**f**) are the average of (**a**,**b**,**d**,**e**), respectively, weighted so that the average intensity in the *x*- and *y*-polarised images is the same.

Having experimentally verified the low-reflection and diffusion properties of random-phase GSP-based metasurfaces, we now centre on the statistics of the diffused light at the wavelength of 800 nm. Starting with the four-element metasurface, Fig. 6a–c display recorded Fourier images for *x*-, *y*- and un-polarised incident light, together with the associated PDFs of the diffused light within the area bounded by the red dashed circles. The area bounded by the inner dashed circle signifies the specularly reflected light, while the outer circle displays the limit of the NA. Furthermore, it should be noted that due to our inability to directly generate truly unpolarised light, Fig. 6c is in fact the average of Fig. 6a,b, weighted so that the average intensity in both images is the same. In accordance with the theoretical predictions [equation (5)], we see that the statistics of the scattered light can quite accurately be described by a negative exponential fit (red solid line) independent of the incident light being linearly polarised or unpolarised. That said, it is clear that the largest discrepancy between recorded statistic and theory occurs for unpolarised light (Fig. 6c), where the close-to-zero intensity values are underrepresented in the experiment. We attribute this observation to lack of fully correlated reflection phases for orthogonal polarisations, owing to the non-square nanobricks, and experimental factors, such a imperfect alignment of the optical set-up, causing inevitable decrease in correlation when the statistic for unpolarised light is based on Fourier images from two orthogonal polarisations. Coming full circle, we have also probed the statistics of the diffused light from the sixteen-element metasurface (Fig. 6d–f). In line with the theory, we observe a significantly different statistics for linearly polarised and unpolarised light, with the former showing negative exponential statistics, while the latter features a PDF that quite accurately can be described by equation (6), thus confirming the statistical independence between orthogonal reflection phases.

## Discussion

In this work, we have studied a specific type of metasurface in which the periodically positioned constituents scatter light with random phases, hence causing incident light to diffusely scatter, with a far-field response being most conveniently described by statistical means. Particularly, we have studied the two extreme cases, where the reflection phases for two orthogonal polarisations are fully correlated or statistically independent, thus leading to statistics that are alike or different for linearly polarised and unpolarised light, respectively. As a way of realisation, we have devised a strategy to make such functionalities in reflection at optical wavelengths by utilising GSP-based metasurfaces, with presented proof-of-concept experiments convincingly verifying the theoretical and numerical predictions. We note that the speckle-like pattern, arising from the diffusion of the incident light, is conventionally realised by (on a wavelength-scale) rough surfaces, but by utilising the new possibilities of light control with metasurfaces we facilitate similar functionality in a very controlled way and on a deeply subwavelength scale. Moreover, we foresee the application of random-phase metasurfaces as high-quality reference structures for dark-field microscopy, hereby avoiding the usage of rather ill-defined scatterers, like dust particles^42^. In a different area of application, we envision that the low-reflection and diffusion of light from random-phase metasurfaces might stimulate interest within the field of camouflage technology, with stealth technology being one example for the low-frequency regime^25^,^26^,^27^. Furthermore, the possibility to impose different statistics on the complex scattered light for linearly polarised and unpolarised light (or, alternatively, for two orthogonal polarizations) renders such metasurfaces interesting for security applications. Also, by incorporating certain correlation between neighbouring metasurface constituents new types of functionalities can be achieved. For example, random-phase metasurfaces encompass the means to realise a new type of Lambertian reflectors. Finally, it is worth mentioning that the current use of plasmonic metasurfaces entails a certain loss of incident power through Ohmic heating. Noting, however, that diffusion of light is achieved through pure phase engineering, it is evident that those losses can be redeemed by utilising dielectric nanoparticles^11^, with a straight forward extension to all-dielectric metasurfaces working in transmission^43^,^44^.

## Methods

### Far-field response of random-phase metasurfaces

The numerical calculations of the far-field response of (ideal and GSP-based) random-phase metasurfaces are all based on the electric far-field defined in equation (7) for *x*-polarised incident light, while a similar expression can be found for *y*-polarised light. As the far-field from any source locally behaves as a plane wave, the time-averaged Poynting vector in the direction 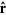 is 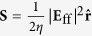, where *η* is the wave impedance of the surrounding medium. The power reflected into the upper half-space (limited by the NA) can be found by direct integration of the power flowing through the sphere segment *r*^2^ sin *θ*d*θ*d*φ*, i.e.,


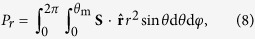


where (*θ*, *φ*) are the conventional polar and azimuthal angles in a spherical coordinate system, and *θ*_*m*_ = sin^−1^ (NA). The effective reflectivity is calculated as *R* = *P*_*r*_/*P*_*i*_, with 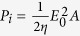 being the power of the incident plane wave on the metasurface of size *A*. We note that the above calculation is implemented in Matlab, ver. 2015a. The uniform random distribution of reflection phases (i.e., unit cells) within the metasurfaces is realized by using either *rand* (for continuous random phase) or *randi* (for discrete random phase) in Matlab, with seeding (controlled by the function *rng*) always being 1. Only in the calculation of Fig. 2d, we use seeding 2 for *y*-polarised incident light in order to mimic the statistical independence between reflection phases of orthogonal polarisations. It should be noted that the response for unpolarised light is obtained by taking the average of the far-field intensities for *x*- and *y*-polarised incident light.

### Simulations

The full-wave simulations are performed using the commercial finite element software Comsol Multiphysics, ver. 5.1. In the calculations, we only model a single unit cell by applying periodic boundary conditions on the vertical sides of the cell. The incident wave is in all cases assumed to be a plane wave propagating normal to the surface with the polarization being either *x*- or *y*-polarised. In our gold-glass-gold configurations, the permittivity of gold is described by interpolated experimental values^45^ while glass, assuming to be silicon dioxide, takes on the constant refractive index 1.45. The medium above the nanobricks is chosen to be air. The air domain is truncated using a port boundary that is transparent for the reflected light, while a perfect electric conductor boundary condition is applied on the bottom side of the optically thick gold substrate. Regarding the complex reflection coefficients (Fig. 3b), the phase is determined at the top surface of the nanobricks.

### Fabrication

The metasurfaces are fabricated on a silicon substrate onto which successive layers of 3 nm Ti, 100 nm Au, 4 nm Ti and 40 nm SiO_2_ are deposited using electron-beam evaporation (metals) and RF-sputtering (SiO_2_). The layer stack is patterned with nanobricks according to the (random) design over an area of 75 × 75 *μ*m^2^ using electron-beam lithography (30 kV acceleration voltage) on a ~100 nm thick PMMA film (950 K A2) followed by development in a solution of IPA:MIBK (3:1), electron-beam deposition of 4 nm Ti and 40 nm Au and finally lift-off in acetone.

### Experimental characterisation

#### Reflection spectra measurement

Refection spectra of the fabricated samples are studied using a home-made linear reflection spectroscopy setup, which includes a microscope (Olympus) and a fiber-coupled spectrometer (Ocean Optics). The reflected light is collected with different objectives and normalized to the corresponding intensity of the beam reflected from a silver mirror.

#### Fourier images

In order to get the Fourier images, the sample is mounted on a stage with *XYZ* translation and exposed to the laser beam from a tunable Ti: Sapphire laser with wavelength set to be 800 nm (for details, see Supplementary Fig. S3). At the same time, the sample can be illuminated with white light for visualization purposes. The polarization state of the incident light is controlled by two polarizers and a half-wave plate. Once the polarization state is fixed, the light is weakly focused by a lens onto the sample with a spot smaller than the metasurface. The reflected light is collected by a long working distance objective, whose numerical aperture is 0.55. The front focal plane is located at the surface of the sample. The diffusion property of the metasurface is finally obtained by projecting the back focal plane of the objective by another lens onto a CMOS camera with high sensitivity.

## Additional Information

**How to cite this article**: Pors, A. *et al.* Random-phase metasurfaces at optical wavelengths. *Sci. Rep.* **6**, 28448; doi: 10.1038/srep28448 (2016).

## Supplementary Material

Supplementary Information

## References

[b1] Pendry J. B. Negative refraction makes a perfect lens. Phys. Rev. Lett. 85, 3966–3969 (2000). 11041972 10.1103/PhysRevLett.85.3966

[b2] Shelby R. A., Smith D. R. & Schultz S. Experimental verification of a negative index of refraction. Science 292, 77–79 (2001). 11292865 10.1126/science.1058847

[b3] Pendry J. B., Schurig D. & Smith D. R. Controlling electromagnetic fields. Science 312, 1780–1782 (2006). 16728597 10.1126/science.1125907

[b4] Schurig D. *et al.* Metamaterial electromagnetic cloak at microwave frequencies. Science 314, 977–980 (2006). 17053110 10.1126/science.1133628

[b5] Fang N., Lee H., Sun C. & Zhang X. Sub–diffraction-limited optical imaging with a silver superlens. Science 308, 534–537 (2005). 15845849 10.1126/science.1108759

[b6] Cai W. & Shalaev V. Optical metamaterials (Springer, New York, 2010).

[b7] Yu N. *et al.* Light propagation with phase discontinuities: Generalized laws of reflection and refraction. Science 334, 333–337 (2011). 21885733 10.1126/science.1210713

[b8] Yu N. & Capasso F. Flat optics with designer metasurfaces. Nat. Mater. 13, 139–150 (2014). 24452357 10.1038/nmat3839

[b9] Estakhri N. M. & Alù A. Recent progress in gradient metasurfaces. J. Opt. Soc. Am. B 33, A21–A30 (2016).

[b10] Pozar D. M., Targonski S. D. & Syrigos H. Design of millimeter wave microstrip reflectarrays. IEEE Trans. Antennas Propag. 45, 287–296 (1997).

[b11] Yang Y. *et al.* Dielectric meta-reflectarray for broadband linear polarization conversion and optical vortex generation. Nano Lett. 14, 1394–1399 (2014). 24547692 10.1021/nl4044482

[b12] Moreau A. *et al.* Controlled-reflectance surfaces with film-coupled colloidal nanoantennas. Nature 493, 86–89 (2012). 23222613 10.1038/nature11615 PMC3584706

[b13] Pors A., Albrektsen O., Radko I. P. & Bozhevolnyi S. I. Gap plasmon-based metasurfaces for total control of reflected light. Sci. Rep. 3, 2155 (2013). 23831621 10.1038/srep02155 PMC3703605

[b14] Pors A. & Bozhevolnyi S. I. Plasmonic metasurfaces for efficient phase control in reflection. Opt. Express 21, 27438–27451 (2013). 24216965 10.1364/oe.21.027438

[b15] Aydin K., Ferry V. E., Briggs R. M. & Atwater H. A. Broadband polarization-independent resonant light absorption using ultrathin plasmonic super absorbers. Nat. Commun. 2, 1–7 (2011). 10.1038/ncomms1528 22044996

[b16] Nielsen M. G., Pors A., Albrektsen O. & Bozhevolnyi S. I. Efficient absorption of visible radiation by gap plasmon resonators. Opt. Express 20, 13311–13319 (2012). 22714359 10.1364/OE.20.013311

[b17] Hao J. *et al.* Optical metamaterial for polarization control. Phys. Rev. A 80, 023807 (2009).

[b18] Sun S. *et al.* High-efficiency broadband anomalous reflection by gradient meta-surfaces. Nano Lett. 12, 6223–6229 (2012). 23189928 10.1021/nl3032668

[b19] Pors A., Nielsen M. G., Eriksen R. L. & Bozhevolnyi S. I. Broadband focusing flat mirrors based on plasmonic gradient metasurfaces. Nano Lett. 13, 829–834 (2013). 23343380 10.1021/nl304761m

[b20] Chen W. T. *et al.* High-efficiency broadband meta-hologram with polarization-controlled dual images. Nano Lett. 14, 225–230 (2014). 24329425 10.1021/nl403811d

[b21] Zheng G. *et al.* Metasurface holograms reaching 80% efficiency. Nat. Nanotechnol. 10, 308–312 (2015). 25705870 10.1038/nnano.2015.2

[b22] Pors A., Nielsen M. G. & Bozhevolnyi S. I. Analog computing using reflective plasmonic metasurfaces. Nano Lett. 15, 791–797 (2015). 25521830 10.1021/nl5047297

[b23] Pors A., Nielsen M. G. & Bozhevolnyi S. I. Plasmonic metagratings for simultaneous determination of stokes parameters. Optica 2, 716–723 (2015).

[b24] Ni X., Wong Z. J., Mrejen M., Wang Y. & Zhang X. An ultrathin invisibility skin cloak for visible light. Science 349, 1310–1314 (2015). 26383946 10.1126/science.aac9411

[b25] Wang K., Zhao J., Cheng Q., Dong D. S. & Cui T. J. Broadband and broad-angle low-scattering metasurface based on hybrid optimization algorithm. Sci. Rep. 4, 5935 (2014). 25089367 10.1038/srep05935 PMC4120860

[b26] Dong D. S. *et al.* Terahertz broadband low-reflection metasurface by controlling phase distributions. Adv. Opt. Mater. 3, 1405–1410 (2015).

[b27] Gao L.-H. *et al.* Broadband diffusion of terahertz waves by multi-bit coding metasurfaces. Light Sci. Appl. 4, e324 (2015).

[b28] Shen Y. *et al.* Phase random metasurfaces for broadband wide-angle radar cross section reduction. Microwave Opt. Technol. Lett. 57, 2813–2819 (2015).

[b29] Su P., Zhao Y., Jia S., Shi W. & Wang H. An ultra-wideband and polarization-independent metasurface for rcs reduction. Sci. Rep. 6, 20387 (2016). 26864084 10.1038/srep20387 PMC4750059

[b30] Paquay M., Iriarte J.-C., Ederra I., Gonzalo R. & de Maagt P. Thin amc structure for radar cross-section reduction. IEEE Trans. Antennas Propag. 55, 3630–3638 (2007).

[b31] Simms S. & Fusco V. Chessboard reflector for RCS reduction. Electron. Lett. 44, 316–317 (2008).

[b32] Chen P.-Y., Soric J. & Alù A. Invisibility and cloaking based on scattering cancellation. Adv. Mater. 24, OP281–OP304 (2012). 23080411 10.1002/adma.201202624

[b33] Andryieuski A., Lavrinenko A. V., Petrov M. & Tretyakov S. A. Homogenization of metasurfaces formed by random resonant particles in periodical lattices. Phys. Rev. B 93, 205127 (2016).

[b34] Albooyeh M., Morits D. & Tretyakov S. A. Effective electric and magnetic properties of metasurfaces in transition from crystalline to amorphous state. Phys. Rev. B 85, 205110 (2012).

[b35] Rockstuhl C. & Scharf T. Amorphous Nanophotonics (Springer Science & Business Media, New York, 2013).

[b36] Albooyeh M. *et al.* Resonant metasurfaces at oblique incidence: interplay of order and disorder. Sci. Rep. 4, 4484 (2014). 24670919 10.1038/srep04484 PMC3967200

[b37] Orfanidis S. J. Electromagnetic Waves and Antennas, URL www.ece.rutgers.edu/orfanidi/ewa (Rutgers University, 2010).

[b38] Goodman J. W. Statistical Optics (Wiley-Interscience, New York, 2000).

[b39] Pors A. & Bozhevolnyi S. I. Gap plasmon-based phase-amplitude metasurfaces: material constraints [invited]. Opt. Mater. Express 5, 2448–2458 (2015).

[b40] Yuan H.-K. *et al.* A negative permeability material at red light. Opt. Express 15, 1076–1083 (2007). 19532335 10.1364/oe.15.001076

[b41] Chen K.-P., Drachev V. P., Borneman J. D., Kildishev A. V. & Shalaev V. M. Drude relaxation rate in grained gold nanoantennas. Nano Lett. 10, 916–922 (2010). 20128610 10.1021/nl9037246

[b42] Fu Y. H., Kuznetsov A. I., Miroshnichenko A. E., Yu Y. F. & Luk’yanchuk B. Directional visible light scattering by silicon nanoparticles. Nat. Commun. 4, 1527 (2013). 23443555 10.1038/ncomms2538

[b43] Decker M. *et al.* High-efficiency dielectric huygens’ surfaces. Adv. Opt. Mater. 3, 813-820 (2015).

[b44] Lin D., Fan P., Hasman E. & Brongersma M. L. Dielectric gradient metasurface optical elements. Science 345, 298–302 (2014). 25035488 10.1126/science.1253213

[b45] Johnson P. B. & Christy R. W. Optical constants of the noble metals. Phys. Rev. B 6, 4370–4379 (1972).

