# Supplementary material for: Random-phase metasurfaces at optical wavelengths
Source: Sci Rep. 2016 Jun 22;6:28448. doi: 10.1038/srep28448 (PMC4916468; doi:10.1038/srep28448)
Supplement: Supplementary Information [file srep28448-s1.pdf]

Supplementary information for

Random-phase metasurfaces at optical wavelengths

Anders Pors,[1,*] Fei Ding,[1] Yiting Chen,[1] Ilya P. Radko,[1,2] and Sergey I. Bozhevolnyi[1]

[1] *SDU Nano Optics, University of Southern Denmark,*
Campusvej 55, DK-5230 Odense M, Denmark

[2] *Department of Physics, Technical University of Denmark, DK-2800 Kongens Lyngby, Denmark*

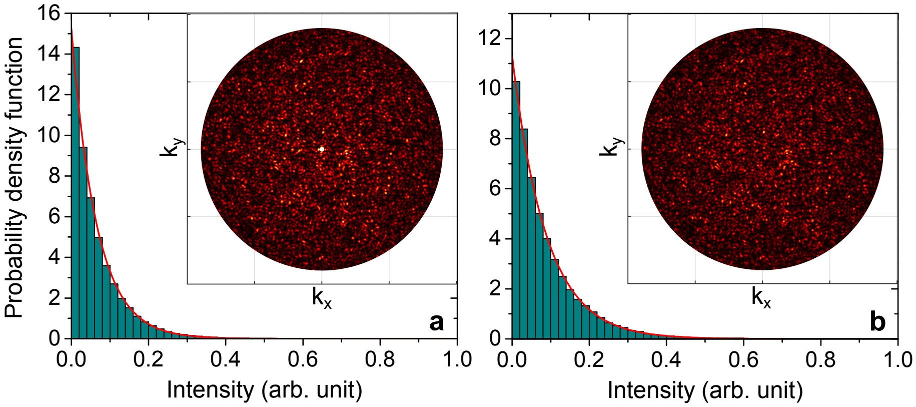

Supplementary Figure S1. **Scattering from random-phase GSP-based metasurfaces with fully correlated reflection coefficients for orthogonal polarisations.** (**a**) Calculated (bar plot) and theoretical (red line) PDF of the far-field intensity (when neglecting specular reflection) from an array of 200×200 unit cells for excitation by unpolarised incident light when the four types of nanobrick unit cells (defined in the main text) appear with equal probability in the array. The inset shows the associated Fourier image of the far-field intensity within the NA$= 0.9$. The image is oversaturated in order to visualise the diffusion of light. (**b**) Similar calculation as in **a**, but the frequency of occurrence of the four nanobricks is scaled by the inverse of their reflection amplitudes. The wavelength is 800 nm.

---

* Correspondence and requests for materials should be addressed to A. P. (alp@iti.sdu.dk)

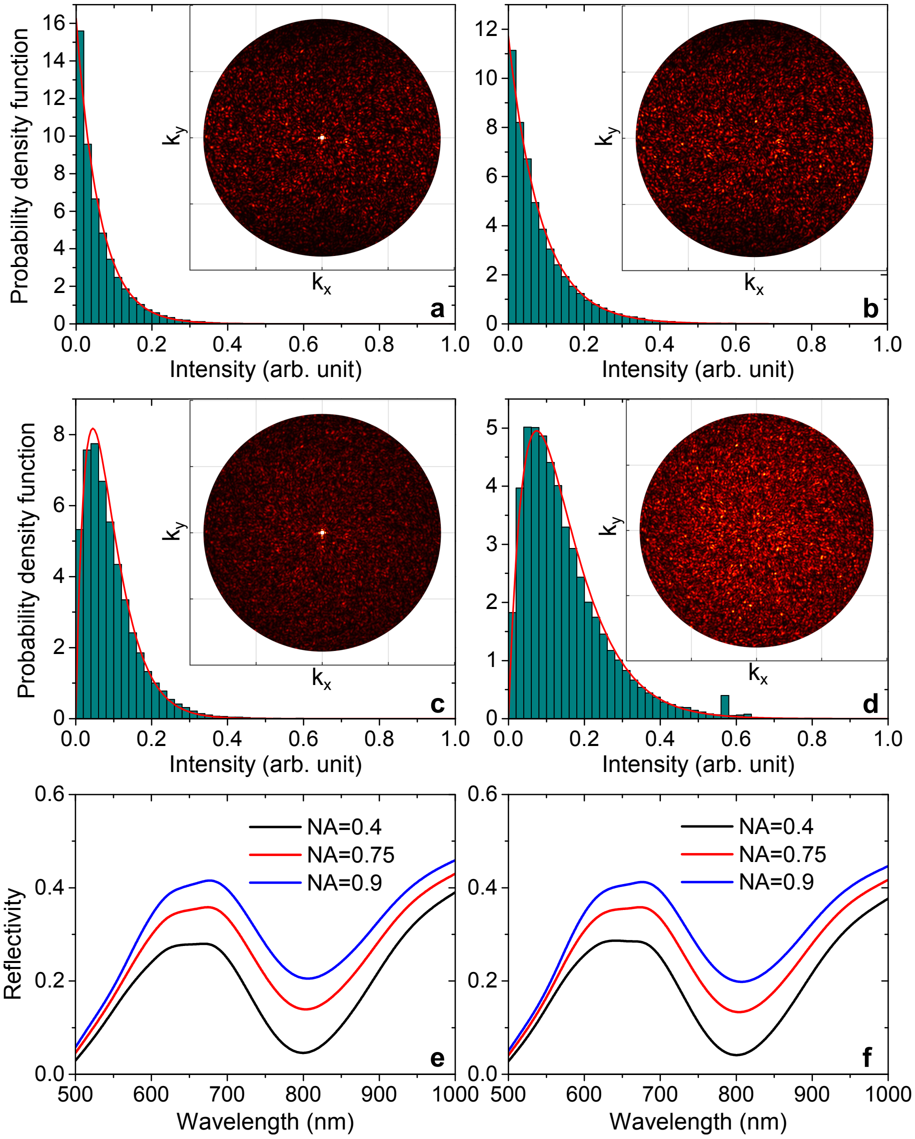

Supplementary Figure S2. **Scattering from random-phase GSP-based metasurfaces with statistically independent reflection coefficients for orthogonal polarisations.** (**a**) Calculated (bar plot) and theoretical (red line) PDF of the far-field intensity (when neglecting specular reflection) from an array of 200×200 unit cells for excitation by x-polarised incident light at $\lambda = 800\,$nm when the sixteen types of nanobrick unit cells (defined in the main text) appear with equal probability in the array. The inset shows the associated Fourier image of the far-field intensity within the NA$= 0.9$. The image is oversaturated in order to visualise the diffusion of light. (**b**) Similar calculation as in **a**, but the frequency of occurrence of the sixteen nanobricks is scaled to compensate for their different scattering strengths. (**c**, **d**) Same calculations as in panels **a** and **b**, respectively, but for unpolarised incident light. (**e**, **f**) Reflectivity as a function of wavelength and NA for the metasurfaces in panels **a** and **b**, respectively. The wavelength-dependence on the reflection coefficients is retrieved from full-wave simulations of the sixteen unit cells.

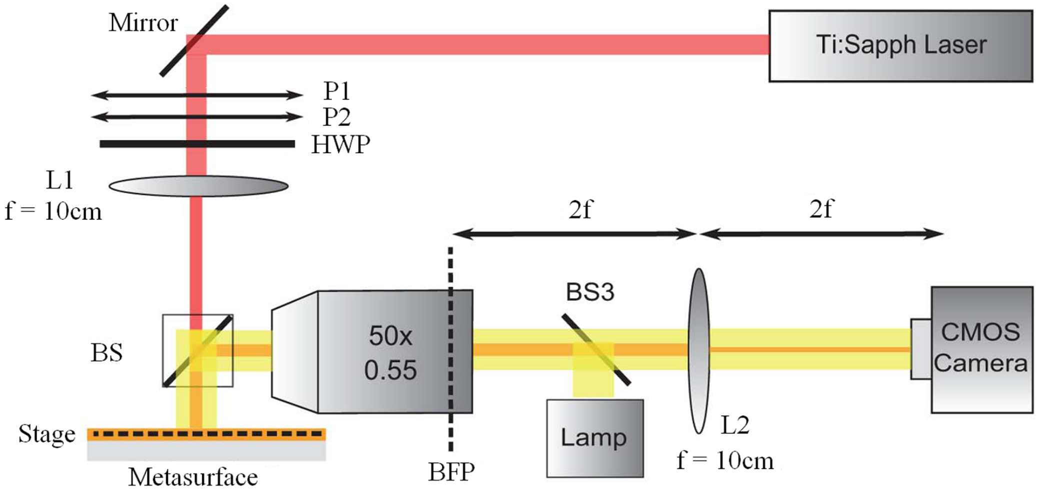

Supplementary Figure S3. **Schematic of experimental setup for optical characterization.** The Fourier images are obtained by mounting the sample on a stage with XYZ translation and exposed to the laser beam from a tunable Ti: Sapphire laser with wavelength of 800 nm. At the same time, the sample can be inspected with white light from a lamp. The polarization state of the incident light is controlled by two polarizers (P1 and P2) and a half-wave plate (HWP). Once the polarization state is fixed, the light is weakly focused by a lens (L1) onto the sample with a spot smaller than the metasurface. The reflected light is collected by a long working distance objective (NA= 0.55). The front focal plane is located at the surface of the sample. The diffusion property of the metasurface is finally obtained by projecting the back focal plane (BFP) of the objective by another lens (L2) onto a CMOS camera with high sensitivity.
